# Extra-Limites

**Published:** 1835-04-01

**Authors:** 


### EXTRA-LIMITES.

### Cholera.

1. The Nature of Cholera Investigated. By J. G. French, M.R.C.S.

2. An Essay on the Use of the Liq. Potassæ and Liquor Alkalinus in the Treatment of Malignant Cholera. By Henry Wilkinson Dodd, M.R.C.S.

The choleraphobia lasted even a shorter time than the epidemic itself. The monster, at a distance, was terrific—and, we must acknowledge that, even when calmly viewed in its just dimensions and proportions, it was a formidable visitor to these peaceful shores. Yet it is astonishing how soon we became familiarised with its presence, and released from inordinate apprehension, when it was actually before our eyes. This philosophic tranquillity was not a little hastened and matured by the assertions of the anti-contagionists, who divested the scourge of more than half its terrors, by stripping it of its preconceived powers of propagating itself from person to person by contact. We believe few, even of our opponents, will deny that this Journal contributed its mite to the consummation of this tranquillity—and still fewer will now raise the cry of contagion, or advocate the system of quarantine and seclusion. But although fear has subsided, all danger is not over. It is probable that the epidemic will return, for a year or two more, in the warm weather, though in gradually diminishing intensity, till it ceases altogether, and shall only be known by history. On this account we shall continue to take brief notice of such publications as may come forth on the subject of cholera.

A great portion of Mr. French's pamphlet is occupied with physiological and pathological discussions on the cause and nature of the disease. The whole theory may be condensed into the following two or three lines. "Thus then we have endeavoured to shew that cholera is produced by a poison, the specific effect of which is to paralyze the heart." If then, says our author, paralysis of the heart be the disease, and the removal of the cause be out of our power, what is to be done? Arguing on its analogy with other acute paralyses, he proposes to diminish the quantity of the circulating fluid, and to effect changes in the blood that remains.

We need not follow our author any farther. We conceive that he has taken a wrong view of the disease. We know nothing of the primary cause; but we know that its first effect is to cause a *serous* hæmorrhage from the stomach and bowels, by which loss of the serous part of the blood, the remainder becomes black and thick—and *then* the heart is unable to circulate the fluid. The patient then dies in the collapse; or, if the hæmorrhage ceases, and the heart is unable to carry on a languid circulation till re-action takes place, we have the choleric fever. Stop this serous hæmorrhage in time and you will save 99 out of the 100 cholera cases. Mr. French's pamphlet is, however, well written, and the reasoning is very ingenious.

Mr. Dodd, a medical practitioner in Durham, informs us that he has been very successful with a particular remedy, and has properly communicated that remedy and the mode of using it, to the public. Mr. D. does not believe that cholera can be communicated from one individual to another. He thinks it was "originally borne to this country by a column of infected matter, and has again been communicated from district to district, by the same means of progression." We doubt this theory, though we cannot disprove it. We think it much more probable that the cause of cholera, whatever it was, originated on the spot where it shewed itself. The circumstance of its inability to cross the bridge of Sunderland for a long time, is a stumbling-block to more than one theory. But leaving this subject altogether, we come to the treatment. Our author, after some ineffectual attempts to check cholera by the common means, came to the resolution of trying the *liquor potassæ*, on the principle of neutralizing some acid in the primæ viæ. The dose was usually about twenty drops in white of egg, repeated in larger quantities, if necessary, till the disease was subdued. Latterly he made use of a composition which he terms " LIQUOR ALKALINUS," composed of eight parts of the liquor potassæ, twelve of " liquor sodæ," and four compound spirit of lavender. The liq. sodæ he made in the same way as the College directs the liq. potassæ to be made. The dose of this compound is larger than that of the liq. potassæ alone. Our author assures us " that the *liq. potassæ and liq. alkalinus* have never yet failed in checking the ricy dejections and ejections of cholera." It generally forms a concrete mass with white of egg, and from 20 to 60 drops were given for a dose. After the discharges of serum from the stomach and bowels were arrested by the medicine, two or three grains of calomel were given every hour until a dark viscid stool was produced. He prevented the patients from taking cold water, for which there is such avidity. We hope the remedy will be tested, should the disease recur in this country.

---

### Horne's Acetum Opii Sedativum.

*To the Editors of the Med. Chir. Review.*

Gentlemen.—I shall feel obliged by your publishing in your valuable Review the mode of preparing the " ACETUM OPII SEDATIVUM," since the means I have pursued for giving it publicity have in part failed, scarcely a day passing without my being requested to point out where it can be procured. The formulary is as follows. I take three times the quantity of opium, ordered by the London Pharmacopœia for making two pints of tincture, mix the opium into a paste with two pints of distilled vinegar and set it by for a few hours; then add a sufficient quantity of alcohol, to extract the remaining virtues of the opium, macerate, and filter. Then introduce the liquor into a retort and distil off the whole of the spirit, (which is saved) and the product in the retort is the required preparation,

There are many practitioners who have conceived that I have " arrogated to myself the fond hope" of placing the acet. op. sed. in competition with morphine. In reply I may briefly state that my first object in introducing it was to endeavour to obtain for the sedative treatment in mania melancholia or puerperal mania, some stronger proof, than simply the detail of a few successful cases, without which, few practitioners would perhaps from my recommendation have heeded it. But now I can state, and " have proofs to prove," that many eminent physicians in this metropolis, and in many distant parts of the country are giving both a fair trial. The second object was either to elicit from Mr. Battley, the method for making his long promised liquor opii sedativus, or to do away with it altogether. The latter object, I believe, will be accomplished; for not one medical gentleman, as yet, who has favoured me by prescribing mine, but has given it the decided preference. Professor Thompson, in the course of a few days, will communicate the results of his experiments in the laboratory and at the bed side.

There are various objections that I might (had I been inclined) urge against the preparations of morphine mostly in use; but mark, not against the action of morphine. 1st. Dr. Turner states " To procure the acetate of morphia in the solid state, it must be evaporated to dryness, and in this process some of its acid is usually expelled. It is deliquescent, and is hence with difficulty preserved in a constant state of dryness; and when neutral it is decomposed by water, whereby part of the morphia is rendered insoluble. In fact, the best mode of employing the acetate is to dissolve given weights of morphia in dilute acetic acid, and preserve it in that form, taking care that the acid is in excess." Query, is not this my preparation? without the trouble or expence of making morphia, and as there are but few manufacturers of it, so it is in proportion adulterated, for it may be procured at the wholesale druggists for 15s. per oz. and for two guineas per oz.

I am gentlemen,
Your obedient and obliged
Servant,
J. H. Horne, Surgeon, &c.
5, *Gerrard Street, Soho,*
20*th February*, 1835.

---

### Advantages of Medical Societies.

In the last annual report of the Hunterian Society (now held at No. 4, Blomfield-street, Finsbury), there are some excellent observations on the benefits which result to the members of medical societies, by periodical associations and free discussions. We shall here present an extract, concurring fully, as we do, in the truth and justice of the remarks.

"The association of medical men for purposes of free discussion has had the sanction of the highest authorities in the profession;—of those who have felt the necessity of maintaining the spirit of observation and research. It has had the sanction of those who have appreciated the importance of concentrating cases, and of submitting them to candid examination, and of bringing opinions under free comparison, that error may be detected, mystery unravelled, and accuracy established.

The successive Reports of this Society have adverted to these advantages, and it is with no small pleasure that your Council, on this occasion, can bear testimony that the unrestrained association of practitioners, in all branches of medical occupation, for conversation on questions of science, arising out of daily intercourse with the sick and injured, in hospitals, dispensaries, and in private life, continues to be felt, in an increasing degree, from the Society's meetings."

"Those who have attended the meetings have often realized the utility of social inquiries in this respect. The correction of error, and the consolidation of truth, have been interwoven with the dissemination of knowledge. The results of researches, bearing on the same point, but conducted by different hands, and under variety of circumstances, have been compared and imbodied, and have left a sequence of caution or of confidence in the application of doubtful remedies." "Sterile hypotheses, and fanciful speculations, have been shunned, but there has been no avoidance of sound theories, nor any prohibition of that freedom of discussion which, whilst it recognizes the basis on which medical opinions can only be safely built, points out defects in admitted theories, or suggests some clearer explanation of the phenomena of disease.

In this way, pre-eminently, is acquired a facility of associating facts, some having shades of difference, but essentially the same; and of discriminating those in which, with some analogy, there are essential discrepancies.

It has often been found highly satisfactory, in the perplexities and responsibilities of practice, to take advantage of the law of combination. The principles which actuate the physician have been combined with those which actuate the surgeon, and one department has thrown light upon the other. A doubt not explicable by the mind in which it originated, but apparently augmenting by the train of anxious thought to which it gave occasion, often receives sudden dispersion on being communicated to another. A mere hint not unfrequently sheds a flood of light, which is like the bursting forth of the sun through a dense cloud. The path which before was intricate and perilous, now lies open to the traveller; surrounding objects have a new aspect; the inward emotions assume a more joyful character, and the intellect is inspired with fresh energy for successful action."

We have wondered at the apathy evinced by many members of the profession in their unwillingness to join in these associations. For our own parts, we can safely aver that, after nearly 40 years of study and experience, in all departments of our profession, we never leave a medical society (and we are members of several) without having made some acquisition to our stock of knowledge. We pity those who think that, after being a few years settled in practice—and especially after having acquired reputation and riches—they are merely wasting time, or acting *infra dignitatem,* if they appear among their brethren, particularly their junior brethren, to communicate and receive information—to discuss different points of theory and practice, and to have the rough angles of self-sufficiency ground down by intercourse with their equals or superiors. We tell these MAGNATES that they do not know their own interest, nor consult the interests of their brethren. If they are wiser or more experienced than their neighbours, they ought to be proud and happy to communicate the overplus of their knowledge to the many needy seekers after information :—if they are below par, they would evince wisdom in *listening*, if they have not the power or the inclination to dispense the fruits of their experience. The very circumstance of becoming personally acquainted with our brethren is no mean advantage accruing from these associations ; and therefore do we earnestly exhort the members of our profession to avail themselves of the benefits they confer, and contribute their support to the institutions in their neighbourhood.

NEW SCHOOL OF ANATOMY IMMEDIATELY ADJOINING ST. GEORGE'S HOSPITAL.

A notice of the opening of this school for anatomical lectures and demonstrations will be found in the Intelligence department of the present number. As the Junior Editor is one of the lecturers of the establishment, he may perhaps be permitted to observe to the country readers of this Journal, that every arrangement has been made to ensure the comforts and preserve the health of the pupils. The dissecting-room is one of the most spacious, the best lit, and best ventilated in London, or perhaps in any Country; and the various accommodations are such as are worthy of the extensive hospital to which it is contiguous. No expense has been, and no pains will be, spared to secure the conveniences, and promote the advancement of those gentlemen who may honour the lecturers with their attendance in the ensuing season.

